# Supplementary material for: Prognostic implication of dynamic platelet count in lung cancer patients with thrombocytosis: a retrospective analysis
Source: PeerJ. 2025 Jun 17;13:e19551. doi: 10.7717/peerj.19551 (PMC12180448; doi:10.7717/peerj.19551)
Supplement: Supplemental Information 2 [file peerj-13-19551-s002.docx]

| **Variable** | **β (95% CI)** | **SE** | **Z** | ***P*** |
| --- | --- | --- | --- | --- |
| **Group (Elevated Platelet)** | -0.339 (-0.632, -0.045) | 0.150 | -2.264 | 0.024 |
| **Sex (Female)** | 0.429 (0.103, 0.755) | 0.166 | 2.579 | 0.01 |
| **Age** | 0.006 (-0.009, 0.022) | 0.008 | 0.801 | 0.423 |
| **MPV** | 0.134 (-0.003, 0.27) | 0.070 | 1.922 | 0.055 |
| **PDW** | 0.049 (-0.013, 0.111) | 0.032 | 1.537 | 0.124 |
| **Lymph** | -0.008 (-0.245, 0.23) | 0.121 | -0.063 | 0.95 |
| **NEU** | -0.033 (-0.082, 0.016) | 0.025 | -1.329 | 0.184 |
| **D-dimer** | -0.05 (-0.089, -0.011) | 0.020 | -2.540 | 0.011 |
| **NLR** | -0.038 (-0.081, 0.006) | 0.022 | -1.680 | 0.093 |
| **PLR** | -0.001 (-0.002, 0) | 0.001 | -1.790 | 0.073 |
| **Histology** |  |  |  |  |
| AC | Reference | - | - | - |
| SCC | -0.037 (-0.462, 0.388) | 0.217 | -0.171 | 0.864 |
| SCLC | -0.688 (-1.022, -0.355) | 0.170 | -4.044 | <0.001 |
| NSCLC | -0.382 (-1.135, 0.372) | 0.385 | -0.993 | 0.321 |
| **Clinical stage (Ⅳ)** | -0.155 (-0.515, 0.204) | 0.183 | -0.847 | 0.397 |
| **Treatment** |  |  |  |  |
| CT | Reference | - | - | - |
| CCT | 0.401 (0.071, 0.73) | 0.168 | 2.385 | 0.017 |
| TT | 0.87 (0.472, 1.268) | 0.203 | 4.288 | <0.001 |
| TCT | 1.443 (0.923, 1.963) | 0.265 | 5.439 | <0.001 |
| IT ± AT | 1.165 (0.187, 2.144) | 0.499 | 2.336 | 0.02 |
