# Supplementary material for: Prognostic implication of dynamic platelet count in lung cancer patients with thrombocytosis: a retrospective analysis
Source: PeerJ. 2025 Jun 17;13:e19551. doi: 10.7717/peerj.19551 (PMC12180448; doi:10.7717/peerj.19551)
Supplement: Supplemental Information 3 [file peerj-13-19551-s003.docx]

| **Variable** | **β (95%CI)** | **SE** | **Z** | ***P*** |
| --- | --- | --- | --- | --- |
| **Treatment** |  |  |  |  |
| CT | Reference | - | - | - |
| CCT | 0.384 (0.059, 0.708) | 0.165 | 2.319 | 0.02 |
| TT | 0.818 (0.426, 1.211) | 0.200 | 4.086 | <0.001 |
| TCT | 1.424 (0.911, 1.937) | 0.262 | 5.442 | <0.001 |
| IT ± AT | 1.149 (0.151, 2.147) | 0.509 | 2.256 | 0.024 |
| **D-dimer** | -0.047 (-0.084, -0.01) | 0.019 | -2.477 | 0.013 |
| **Group (Elevated Platelet)** | -0.296 (-0.568, -0.023) | 0.139 | -2.123 | 0.034 |
