# Supplementary figures and images for: Prognostic implication of dynamic platelet count in lung cancer patients with thrombocytosis: a retrospective analysis
Source: PeerJ. 2025 Jun 17;13:e19551. doi: 10.7717/peerj.19551 (PMC12180448; doi:10.7717/peerj.19551)

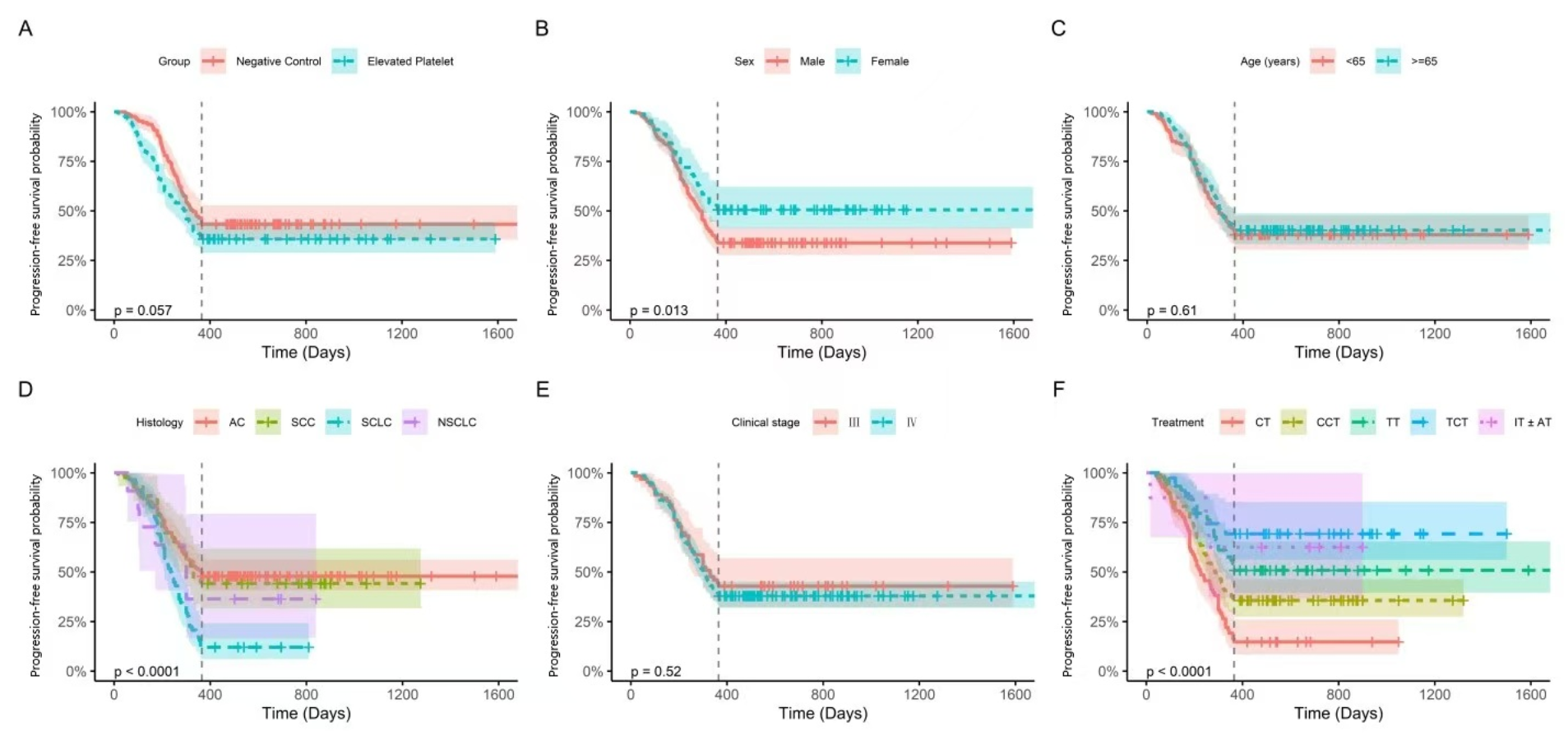

Supplement: Supplemental Information 4 — The vertical line represents time = 365 days, which is the 1-year follow-up point. Panels (A) to (F) display the analyzed variables, including group, sex, age, histology, clinical stage and treatment, respectively. Lymphocytes (Lymph); Neutrophils (NEU); Adenocarcinoma (AC); squamouscellcarcinoma (SCC); small cell lung cancer (SCLC); chemotherapy (CT); chemo-based combination therapy (CCT); immunotherapy (IT); antiangiogenictherapy (AT); targetedtherapy (TT); targeted combination therapy(TCT), the same below. [file peerj-13-19551-s004.png]
